# Research on the dynamic evolution law of fissures in shallow-buried and short-distance coal seam mining in Lijiahao Coal Mine

**DOI:** 10.1038/s41598-023-32849-1

**Published:** 2023-04-06

**Authors:** Beifang Wang, Duo Zhou, Jing Zhang, Bing Liang

**Affiliations:** 1 School of Mines, Liaoning Technical University, Fuxin, 123000 Liaoning China; 2 State Key Laboratory of Water Resource Protection and Utilization in Coal Mining, Beijing, 100011 China; 3 School of Science, Liaoning Technical University, Fuxin, 123000 Liaoning China; 4 School of Mechanics and Engineering, Liaoning Technical University, Fuxin, 123000 Liaoning China

**Keywords:** Engineering, Geology, Petrology

## Abstract

Aiming at the problem of spontaneous combustion of coal relics caused by the overburden fracture network penetrating the upper and lower coal seams in the process of shallow-buried and short-distance coal seam mining, the 31114 working face of Lijiahao coal mine was used as the research background to study the characteristics of overburden transport and fracture development in shallow-buried and short-distance coal seam mining by using physical similar simulation test; the fractal dimension and image processing techniques were used to quantify the overburden fractures; the classical mechanical models of "solid support beam" and "masonry beam" were combined to analyze the causes of fracture dynamic evolution. The results show that: (1) Before the key seam fracture, the stress in the upper rock seam only changes in a small amount, and the stress in the lower rock seam evolves similarly to the single coal seam mining; when the key seam fracture is broken, the stress in the upper and lower rock seams will change by jumps. (2) The fractal dimension of the fissures rised from 1.4 to 1.5, the total area of fissures is increased from 16,638 pixels to 17,707 pixels, and the total length is increased from 2217 to 3071 pixels; after the main key layer of the overlying rock is broken, the fractal dimension of the fissures is reduced from 1.56 to 1.5, and the total area of fissures is reduced from 31,451 pixels to 29,089 pixels, the total length has increased from 5657 to 6619 pixels. (3) Before the key layer between the coal seams is broken, it will be suspended to form a "fixed beam". After the first break, the broken rock above it will settle synchronously until the rock blocks form a hinged structure and then collapse. After the fall stops, the key layer periodically breaks to form a "masonry beam" structure, and the overlying stratum settles synchronously.

## Introduction

Most of the coal seams mined in the Dongsheng mining area in western China are spontaneous combustion and easy spontaneous combustion coal seams, and the coal seams are buried shallowly and have small spacing. In recent years, with the increasing intensity of coal resource mining, the first coal seam mining in each mine has basically ended, and the mining has gradually entered the short-distance goaf. A large number of on-site practices show that in the mining of shallow-buried and short-distance coal seam groups, mining fissures can easily connect the upper coal seam goaf and the lower coal seam working face, and the fissures will develop to the surface and cause mine air leakage, making the coal left in the goaf that poses a hidden danger of spontaneous combustion protrude^1^.

Therefore, it is very important to study the evolution law of fissures in shallow-buried and short-distance coal seam mining for the safety of coal mine production. Many scholars in China and abroad have carried out a series of studies on the evolution law of coal seam overlying fissures, and have achieved a lot of results. Shavarskyi et al.^2^ used numerical software to simulate the stress–strain state of the surrounding rock during the advance of the longwall working face, and concluded that the overburden transport and fracture development were affected by the advance speed. Smoliński et al.^3^ found by numerical simulation tests that the waste rock left in short-distance coal seam bottom rock in the quarry area could reduce the overburden support pressure and its fracture development. Dychkovskyi et al.^4^ analyzed the nature of the support pressure area formation in front of a stope as well as along the extraction pillar length during the double-unit longwall operation by building a 3D simulation model. Vu^5^ focused on 5 main causes and factors ruling face spall and roof falling in longwalls, and proposed some control programs. Academician Qian et al. put forward the key layer theory and the "O" ring theory^6,7^. Academician Liu^8^ established the theoretical system of "horizontal three zones" and "vertical three zones" on the basis of the overlying strata damage and the distribution characteristics of water-conducting fractures. Cheng et al.^9^ obtained the distribution and evolution characteristics of fractures under the influence of double mining of the protective layer and the protected layer through similar simulation experiments. Zhang et al.^10^ constructed a three-dimensional model of a "mining fracture annular body" and gave its boundary discrimination method. Zhao et al.^11^ used similar material simulation experiments to conclude that the development of cracks can be divided into three stages: before the initial roof collapse, during the periodic roof collapse, and near the working face. Wang^12^ obtained the general distribution characteristics of overlying fissures through similar simulation experiments. And after processing the images of the fracture network, the general law of the evolution of the morphological parameters of the fracture network is obtained. Wang et al.^13^ used similar simulation experiments to obtain the characteristic effects of repeated mining of short-range coal seams on overlying rock displacement, mine pressure, and fracture evolution. He et al.^14^ studied the formation of low cantilever beam and high hinge beam structures after the key layer collapses when the ultra-thick coal seam is mined with long-wall mechanized top coal caving. Huang et al.^15^ studied the roof caving characteristics of a large mining height working face through physical and numerical simulation experiments and on-site drilling measurements, and proposed that the caving has significant time and space effects. Due to the complex engineering conditions of coal seam mining, the simplification of the theoretical model often has a great impact on the analysis results. Therefore, many scholars have also paid attention to the actual measurement research of engineering, and have studied the development height and distribution law of coal seam roof cracks through a large number of field observations^16–18^.

After the shallow-buried and short-distance coal seam is mined, the rock formation will inevitably move and break under the influence of mining, forming mining fissures. The leakage of air and oxygen supply in the mining fissures is the main reason for the spontaneous combustion of coal left in the upper and lower gobs of Lijiahao Coal Mine, while a continuous and stable supply of oxygen is a necessary condition for the continuous combustion and development of coal leftover in goaf. However, there is a lack of in-depth research on the dynamic distribution characteristics of mining fissures during rock movement, especially the lack of quantitative description of the dynamic distribution of mining fissures in overlying rocks. Therefore, in order to effectively prevent and control the natural fire of coal relics in the compound mining area of Lijiahao mine, reduce and control the air leakage and oxygen supply in the mining area, the author selects the 31114 working face of Lijiahao Coal Mine as the research object, simulates the overburden migration and fracture development characteristics of the mining face experimentally, and theoretically analyzes the overburden fracture and fracture evolution law of shallow-buried and short-distance coal seam mining, so as to provide the safe production of Lijiahao Coal Mine provide an important theoretical basis.

## Study area

Lijiahao Coal Mine is located in Dongsheng District, Ordos City. It is a typical shallow-buried short-distance coal seam mining mine. The thickness of the loose layer of the coal seam is 10 ~ 50 m, the thickness of the bedrock is 170 ~ 210 m, and it is a single main key layer type. The main coal seams of the mine are 2–2 coal seams and 3-1 coal seams. The average mining height of 2–2 coal seams is 3 m, and all the mining has been completed. At this stage, 3-1 coal seams are being mined, and the average mining height of 3-1 coal seams is 5 m. The full-thickness coal mining method is adopted at one time, and the roof is managed by the natural caving method. The thickness of the two coal seams is stable, the structure is single, and the coal seam dip angle is 0° ~ 3°. The average distance between the 2–2 coal seam and the 3-1 coal seam is 30 m, and there is a key layer between the coal seams and the rock layers. The 2–2 coal seam working face and the 3-1 working face are arranged at a certain distance, and the lengths are different. Therefore, part of the 31114 working face above the mining area is the 2–2 coal seam goaf, and another part is the 2–2 coal seam solid coal. Field practice shows that in the mining process of 3-1 coal seam in Lijiahao Coal Mine, mining-induced fissures easily pass through the working face of 3-1 coal seam and the mining area of 2–2 coal seam and develop to the surface to cause mine air leakage, which leads to the continuous influx of oxygen into the goaf and increases the risk of spontaneous combustion of coal left in the goaf.

## Methods

In order to directly reflect the migration and destruction of the overlying rock, the development and evolution of the fissures in the shallow-buried and short-distance coal seam mining of the Lijiahao Coal Mine, the two-dimensional physical similarity simulation is first used to study.

### Model making

#### Material ratio

River sand and mica are used as aggregates, and lime and gypsum are used as cements to construct a similar simulation model of shallow-buried and short-distance coal seam mining in Lijiahao Coal Mine^19–21^, and the experimental platform is simulated by planar similar materials with length, width and height of 3000 mm, 300 mm and 2000 mm.

The model similarity constant is determined according to the site conditions and the similarity law, in which the geometric similarity ratio is 1:100, the bulk density similarity ratio is 1:1.6, and the time similarity ratio is 1:10. According to the physical and mechanical parameters of the actual coal stratum in the coal mine, the ratio number is obtained by conversion and comparison with the ratio table of similar simulated materials. The physical and mechanical parameters of the rock formation are shown in Table 1.

**Table 1:** Physical and mechanical parameters of rock stratum.

No	Layer	Thickness (m)	Simulated thickness (cm)	Number of layers	Compressive strength (MPa)	Simulated compressive strength (MPa)	Apparent density (g/cm^3^)	Simulated apparent density (g/cm^3^)
1	Fine-grained sandstone	4	4	2	44	0.28	2.7	1.8
2	Sandy mudstone	13.6	14	7	25	0.16	2.55	1.7
3	3-1 Coal	5.7	5	3	22	0.20	2.55	1.7
4	Fine-grained sandstone	26.7	26	13	44	0.28	2.7	1.8
5	Sandy mudstone	2.4	2	1	25	0.16	2.5	1.67
6	2–2 Coal	3.1	3	2	17	0.08	2.55	1.7
7	Sandy mudstone	2.7	3	2	28	0.18	2.45	1.63
8	Coal	2.1	2	1	25	0.16	2.55	1.7
9	Siltstone	1.6	2	1	44	0.28	2.7	1.8
10	Mudstone	8.1	8	4	12	0.08	2.55	1.7
11	Siltstone	8	8	4	20	0.13	2.72	1.81
12	Siltstone	3.2	3	2	18	0.08	2.55	1.7
13	Fine-grained sandstone	28	28	14	21	0.13	2.7	1.8
14	Fine-grained sandstone	2.8	3	2	19	0.08	2.72	1.81
15	Sandy mudstone	3.4	3	2	12	0.20	2.45	1.63
16	Siltstone	12	12	6	25	0.16	2.55	1.7

The thickness of the simulated rock layer obtained from the geometric similarity constant cannot reflect all the histograms, and the method of equivalent stress loading is applied to the overlying rock layer that cannot be stacked. According to the self-weight stress field of the overlying strata, the stress similarity constant is used to convert it into a simulated stress value. The vertical pressure that needs to be loaded onto the model is 15 kPa. In order to monitor the change of vertical stress in the excavated section inside the model in real time, the pressure sensors are pre-buried in the process of laying similar material model, and the sensors are monitored by stress monitor, which is connected to the computer to collect the data on the miniature pressure sensor.

#### Measuring point layout

**Figure 1:**
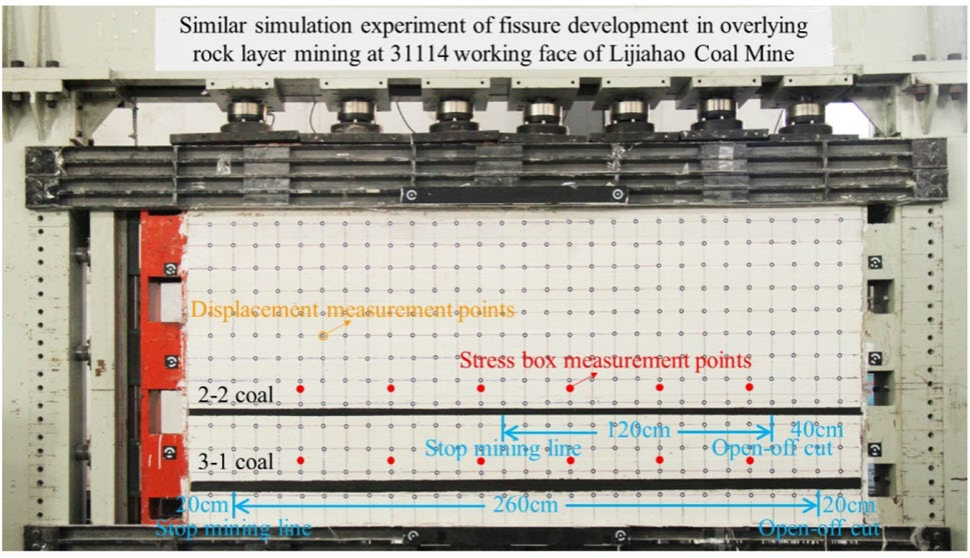
Model laying object.

The coal seam mining will cause displacement and stress redistribution in the rock formation around the mining space. Therefore, in the process of model excavation, the stress and displacement of the overlying rock need to be detected. The vertical and horizontal lines were drawn on the front of the model, and the distance between the horizontal and vertical lines was 10 cm. Non-coding points were set at the intersections of the lines. The XJTUDP three-dimensional optical photogrammetry system was used to observe the displacement changes of the non-coding points of the overlying rock. A stress measurement line parallel to the coal seam was arranged in the rock layers 10 cm above the coal seams of models 2–2 and 3-1, respectively, to detect the real-time changes of the vertical stress of the overlying rock during the coal seam mining process. A total of 12 survey points are arranged in the two survey lines, the number of survey points above the 3-1 coal seam is from 1 to 6, and the number of survey points above the 2–2 coal seam is from 7 to 12. Among them, the distance between the measurement points at both ends of the stress measurement line is 50 cm from the model boundary, and the distance between the adjacent measurement points on each measurement line is 40 cm. Before the model is mined, the value of each stress measurement point must be cleared, that is, the original rock stress of each measurement point must be at the 0 scale line. If the stress value becomes positive during the mining process, it means that the measurement point is in a pressurized state. If it becomes negative, it is in a pressure-relief state. The actual layout of the model is shown in Fig. 1.

### Model mining

According to the actual production situation at Lijiahao Coal Mine, 2–2 coal seams were mined first, the cutting hole was 40 cm away from the right boundary of the model, and the working face was advanced by a total of 120 cm. The 3-1 coal seam will be mined after the overlying rock of the 2–2 coal seam has basically stabilized. The 3-1 coal seam's incision is 20 cm away from the right boundary of the model, and the total advance is 260 cm. The two seams are mined from right to left. Calculated according to the aforementioned size similarity ratio and time similarity ratio, in the test, the two coal seam production teams mined 4 cm each time and every 40 min.

### Experimental results

#### Characteristics of overburden caving and fracture development

**Figure 2:**
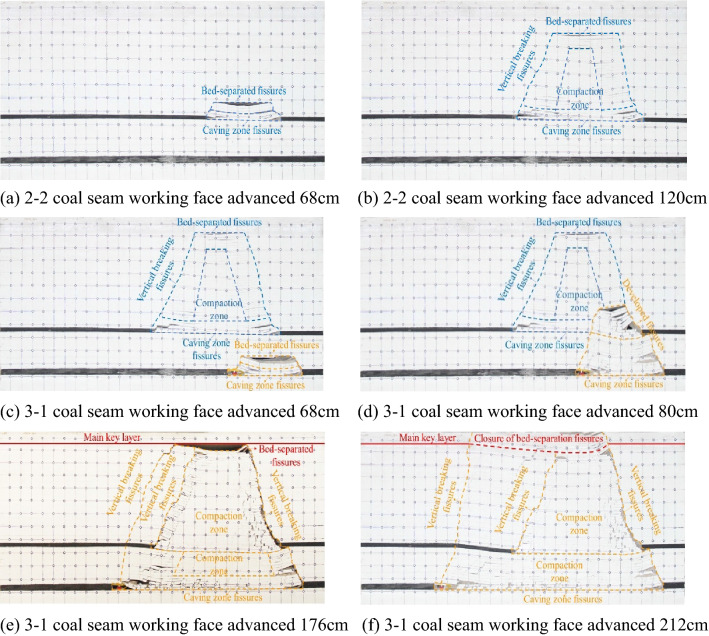
Overburden collapse diagram of working face.

In the mining process, the model has successively experienced three stages: "upper coal seam mining", "gob mining", and "solid coal mining." The "upper coal seam mining" stage is the "2–2 coal seam mining stage," and the characteristics of overburden collapse and fissure development are similar to those of general single coal seam mining. When the basic roof collapses for the first time, the roof rock layer of the goaf begins to separate and collapse under the action of its own gravity and the pressure of the overlying rock layer, forming a caving zone and a fissure zone structure, as shown in Fig. 2a. During the subsequent advancement of the working face, the overlying rock periodically caves in the form of "cantilever—articulation—collapse—stabilisation" and the cracks are periodically generated, closed, and compacted, resulting in the phenomenon of cracks expanding upwards and towards the working face. Every time the overlying rock collapses, a layer-separated fissure zone will be formed above the goaf, the caving fissure zone will be formed at the bottom, and the middle part will be the compaction zone. Above the side of the opening and the stop line, the area where the rock blocks are hinged vertical fissure zone is formed, as shown in Fig. 2b. At the beginning of 3-1 coal seam mining, although the roof did not collapse, the stress balance state of the overall surrounding rock changed due to the mining operation, and the height of the fissure zone in the gob of the upper coal seam was further developed upward. After that, the working face entered the mining stage under the goaf. When it advanced to 68 cm, the roof of the 3-1 coal seam collapsed, and obvious separation cracks were formed above it, as shown in Fig. 2c. When the working face continues to advance to 80 cm, the key layer between the coal seams is broken, the upper and lower coal seam gobs are connected, and the collapsed rock layers in the original 2–2 coal seam gobs will undergo secondary caving, and the cracks are criss-crossed at this time. The shape is extremely complex, as shown in Fig. 2d. During the subsequent advancement of the working face, the key layers between the coal seams were broken many times, the "masonry beam" structure continued to evolve with the advancement of the working face, and the pressure changes in the working face were frequent and periodically reduced. After passing through the upper coal seam goaf, a permanent vertical fracture is formed in the overlying rock above the 2–2 coal seam stop line, as shown in Fig. 2e. After that, the working face resumes periodic pressure, the frequency of pressure decreases, and the overlying rock collapses. The development of falls and fissures has returned to the characteristics of single coal seam mining. When the main key layer of the coal seam bedrock is broken, the layer separation fissure above the fissure zone is closed, the topsoil layer collapses simultaneously, and the fissure penetrates the surface, as shown in Fig. 2f.

#### Stress distribution characteristics

**Figure 3:**
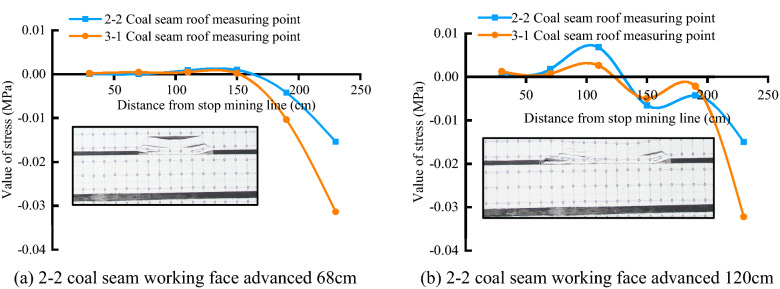
Stress distribution of 2–2 coal seam mining.

**Figure 4:**
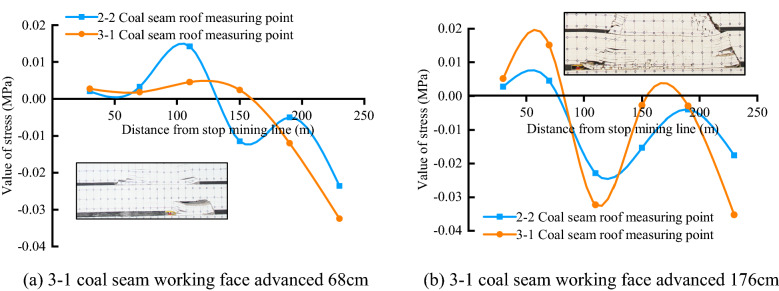
Stress distribution of 3-1 coal seam mining.

The distribution of rock formation stress has been in dynamic change with coal seam mining, and its stress evolution process can also be divided into three stages: the upper coal seam mining stage, the gob mining stage, and the solid coal mining stage. Figures 3 and 4, respectively, show the stress distribution of the overlying rock in the key mining stages of the upper and lower coal seams. The blue line represents the stress distribution of the 2–2 coal seam roof stress line, and the orange line is the 3-1 coal seam roof stress line stress distribution. Comparing Figs. 3 and 4, it can be seen that there is a significant difference between the stress evolution process of short-distance coal seam mining and single coal seam mining.

After the mining of the 2–2 coal seam, the stress value at the measuring point on the side of the goaf decreases rapidly, the advanced bearing stress is concentrated in the rock layer near the coal wall side of the working face, and the stress value at the measuring point increases. When the stress is higher than the yield strength of the rock mass After that, the roof stratum yields and breaks, and the stress value at the measuring point decreases in a leap, and cracks are generated in this process. After the roof rock layer has been fully collapsed and recompacted, the stress value near the collapsed rock layer in the goaf is restored to the original rock stress level, and most of the cracks are also closed at this time. During the whole mining process of the 2–2 coal seam, the stress changes on the roof and floor show three changing processes of and the corresponding areas are: the pressure-boosting area, the pressure-relieving area, the stress-recovery area, and the overall stress after mining. The distribution conforms to the "saddle-shaped" distribution characteristic.

After the mining of the 3-1 coal seam, the overburden stress has further changed from the previous distribution state, but during the mining process of the coal seam under the goaf, the stress values of the upper and lower measurement lines no longer change synchronously. The 3-1 coal seam roof measurement the variation of linear stress is similar to the variation of the stress value of the roof rock stratum in single coal seam mining, but the linear stress value of the 2–2 coal seam roof measurement has unique variation characteristics. Before the key layer between the coal seams is broken, in the overlying rock in front of the 3-1 coal seam working face, the stress value of the measuring point on the orange measuring line changes from a negative value to a positive value, indicating that the stress is concentrated, and after the working face passes the measuring point, the stress value of the measuring point decreases again, but the stress value of the blue measuring line measuring point at the same position does not change significantly during this process. After the key layer is broken and collapsed, the stress value of the upper measuring point suddenly decreases, and the stress value of the lower measuring point suddenly increases, until the working face is far away from the measuring point and the stress value slowly returns to the original rock stress level. After that, every time the key layer collapses, the stress value of the measuring point of the upper secondary caving rock layer will decrease significantly, and the stress value of the measuring point of the lower rock layer will increase. When the 3-1 coal seam working face is close to the 2–2 coal seam stop mining line, the leading stress value of the orange measuring line reaches 0.015 MPa, which has exceeded the stress value of the blue measuring line below 0.004 MPa. At this time, the stress value distribution along the upper and lower measuring lines is also roughly close to "saddle shape". Later, during the mining process of the working face under the solid coal, the stress changes of the upper and lower survey lines returned to the same consistency. It is worth noting that during the whole mining process, the stress value of the measuring point near the open-cut side has been low, which is the same as the low stress value on the side of the gob of the working face because the rock formation here is always in the mining process. It is not compacted, so the rock formation at the measuring point has been in a pressure relief state.

Comparing the similar simulation crack distribution with the roof stress distribution, it can also be seen that the "vertical fracture crack" in the similar simulation results is in the same position as the pressure relief area, and the "caving crack area" is in the same position as the stress recovery area.

#### Characteristics of overburden subsidence

Through the extraction and analysis of the rock stratum displacement measuring point data after the mining of the similar simulation experiment, it can be judged that the mining fractures are mainly enriched in the areas where the displacement values of the adjacent measuring points are significantly different. The displacement vector of the overlying stratum in the abscission fissure area is downward, and the displacement change of the overlying stratum is not synchronous, resulting in a stratum-separated fissure, which is analysed to be mainly generated by rock tension. The vector direction of the displacement field of the rock formation in the compaction area is also downward, and the displacement is basically the same. The displacement of the rock layers on both sides of the vertical fracture is different, and the fracture is generated by the shear fracture of the rock layer.

**Figure 5:**
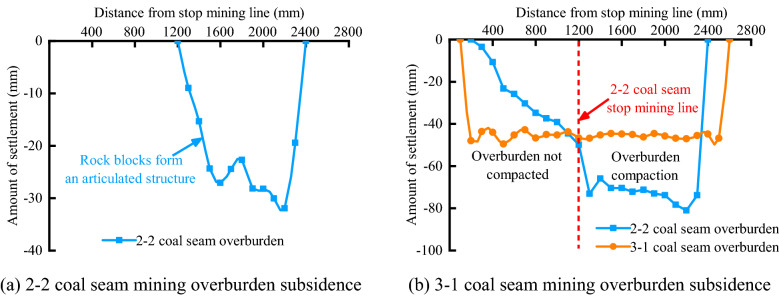
Settlement of overburden rock near the goaf area for close coal seam mining.

Figure 5 shows the settlement of the rock formation near the goaf after the coal seam mining is completed. The abscissa represents the distance between the displacement measuring point in the horizontal direction and the stop line of the 3-1 coal seam working face, and the blue line represents the settlement of the displacement measuring line at 2 cm above the 2–2 coal seam, the orange line represents the settlement of the displacement measuring line at 4 cm above the 3-1 coal seam, and the full length of the 2–2 coal seam working face advancement is between 1200 and 2400 mm on the abscissa. Figure 5a shows that the overlying rocks on the side of the 2–2 seam incision and the side of the mining stop line are in an inclined arrangement, the rock blocks form a hinged structure, and the "vertical fractures" are distributed in this overlying area. After the 3-1 coal seam mining is completed, the overlying rock subsides again, and the subsidence amount of the overlying rock survey line is shown in Fig. 5b. At the two ends of the two displacement lines, the subsidence changes significantly, the rock block rotates obviously, and the vertical fracture area is enriched here. The strata of the 2–2 overlying coal seam are arranged obliquely between 500 and 1200 mm. Combined with similar simulation results, the lower strata in this area have not been fully compacted, but the upper strata are relatively closely arranged, so there is no obvious fracture development in this area. The subsidence value at the stop line of the 2–2 coal seam also has obvious changes. Due to the lack of a coal seam, the overlying rock here cannot be compacted, and the vertical cracks will exist permanently. Comparing Fig. 5a,b, after the mining of 3-1 coal seam is completed, the subsidence values of the strata in the goaf of 2–2 coal seam tend to be the same, indicating that the rock strata in the caving zone of 2–2 coal seam are further compressed. In fact, the number of cracks has decreased.

## Results and discussion

### Quantitative analysis of mining fissures

The development of computers has provided great convenience for the identification and quantification of cracks in images. The use of computers can accurately quantify cracks in pictures and obtain geometric parameters such as the length and area of cracks. Previous studies have shown that the developmental morphology of fissures has high self-similarity, so the fractal dimension can also be used to quantitatively describe fissures^22^. The overlying fissure images obtained in the similar material simulation test are RGB color images. Since the computer can only receive digital information, it is necessary to convert the color image into a binary image so that the computer can be used to further identify and measure the fissures. The obtained binary image the map can also directly reflect the distribution and development of fissures.

#### Binarization of fissure images

**Figure 6:**
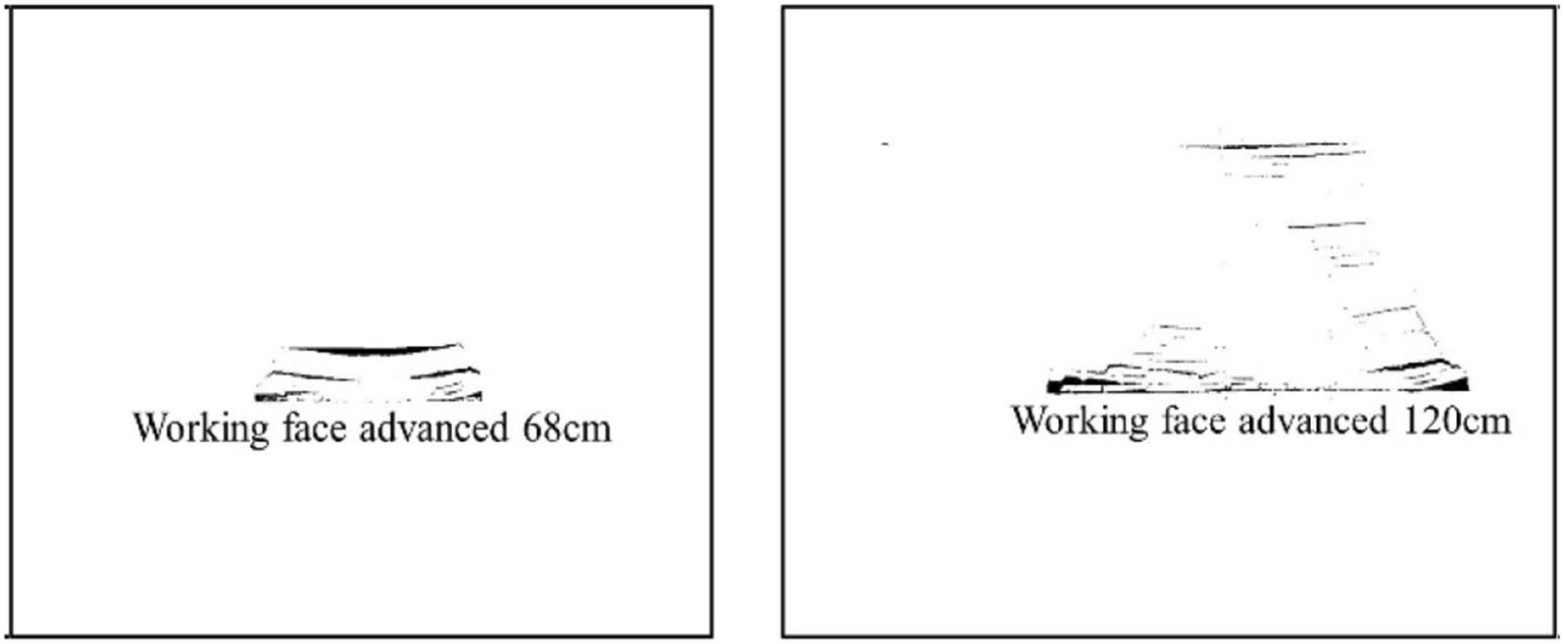
Binary diagram of 2–2 coal seam mining fissure evolution.

**Figure 7:**
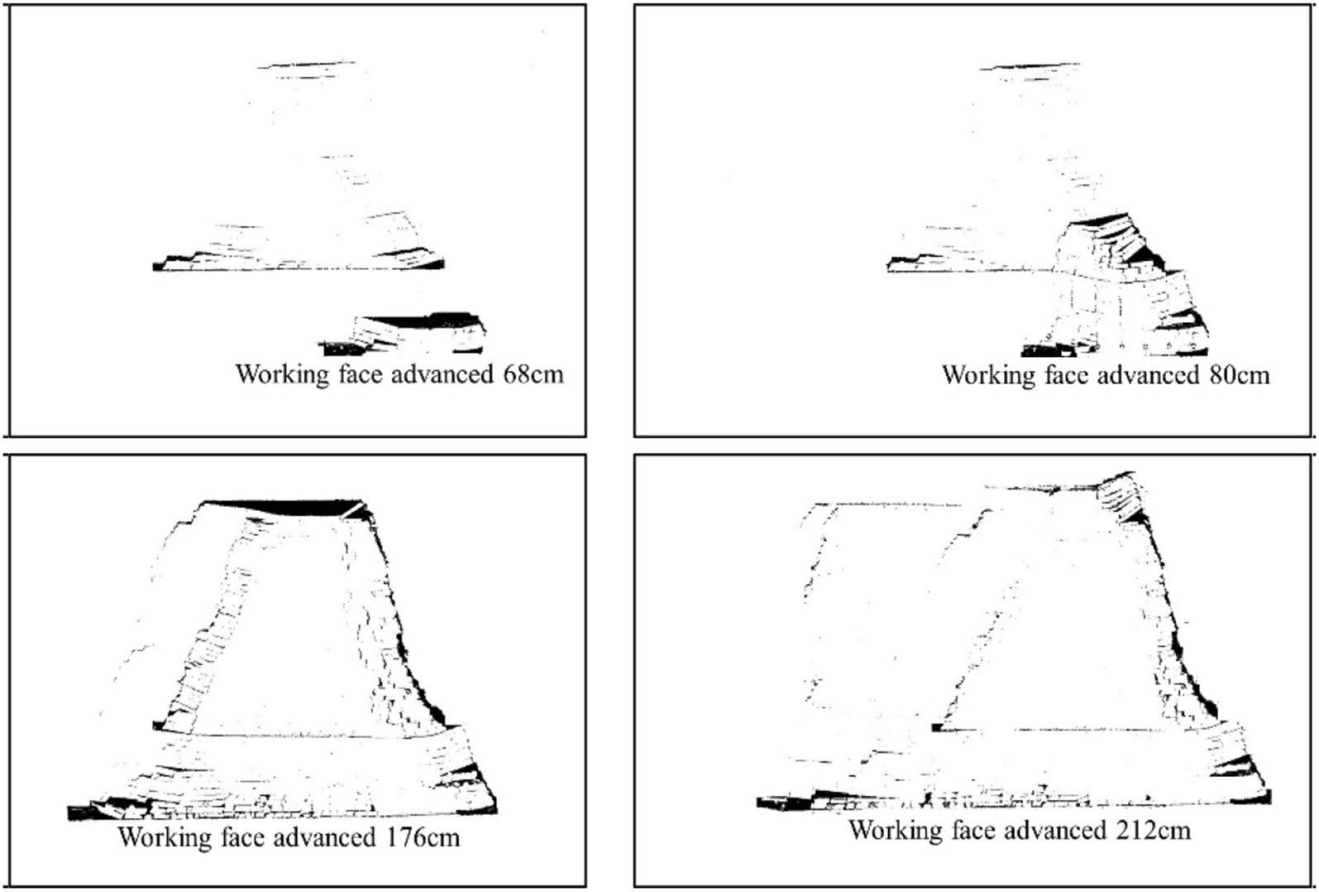
Binary diagram of 3-1 coal seam mining fissure development.

First, convert the color crack image into a grayscale image, then use the homomorphic filtering method to remove the more prominent "noise" in the image, and finally use the threshold segmentation method to convert the image with continuously changing grayscale into two colors with only a black and white binary map^23,24^. The finally obtained partial fracture evolution binary map is shown in Figs. 6 and 7.

#### Fractal dimension, length and area of the overall fissures

In this paper, the most commonly used box-counting dimension is used to quantify the overall fracture of the overlying rock, and the formula is:

1 $$D = \mathop {\lim }\limits_{r \to 0} \frac{\log N(r)}{{\log (1/r)}}$$

where *D* is the dimension of the box; *N* is the number of grids that divide the space; *r* is the side length of the grid.

The principle is to put the cracked binary image on a grid that can be divided evenly, and through the grid step by step fine segmentation, to see the changes in the number of box covers, so as to calculate the box dimension^25–27^. The principle of computer identification of the length and area of cracks is as follows: first, the cracks in the binary image are divided to obtain the crack grid; then, the center axis of the crack is extracted from the crack grid; and finally, the nodes and crack segments of the center axis are identified, and various geometric parameters of the crack grid are calculated. By using these geometric parameters, the length, width, and area of each fracture can be obtained^28,29^. The resulting fracture length and area are in pixels.

#### Fractal dimension, length and area of the overall fissures

**Figure 8:**
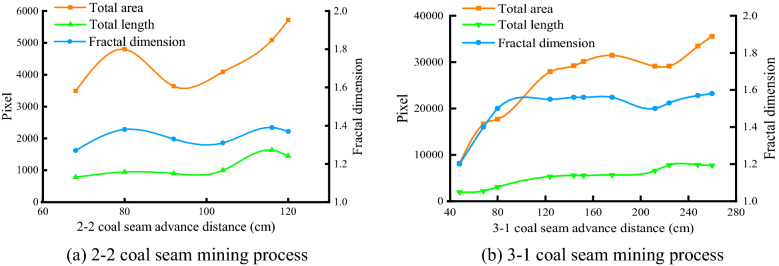
Calculation results of fracture fractal dimension.

Figure 8 shows the relationship between the fractal dimension of the fracture and the length and area of the fracture during the advancing process of the working face. The blue line is the fracture analysis dimension, the orange line is the fracture area, and the green line is the fracture length.

It can be seen from Fig. 8 that, except for individual spanning stages, the fractal dimension of the fracture, the length and area of the fracture—increase or decrease synchronously with the advancement of the working face, showing a strong correlation. In the process of 2–2 coal seam mining, the fracture fractal dimension, total fracture length, and total area curve show a trend of first increasing, then decreasing, and then increasing with the advancement of the working face, and the overall change is relatively gentle. When the basic top of the 2–2 coal seam collapsed for the first time, the fractal dimension of the fissure was 1.27, the length of the fissure was 783 pixels, and the area of the fissure was 3489 pixels, all of which were the lowest values in the whole mining stage. When the overlying rock collapsed for the last time, the fractal dimension of the fracture reached its maximum value of 1.39 in the mining stage, the total length of the fracture was 1635 pixels, and the total area of the fracture was 5083 pixels. The fluctuation of data lines is due to the inherent randomness of fracture development, and the degree of rock layer compaction in each caving stage is different, which makes the development of fissures different in each caving stage.

In the 3-1 coal seam mining stage, when the gobs of the upper and lower coal seams are penetrated, the total length and total area of the fissures increase, the fractal dimension of the fissures increases from 1.4 to 1.5, the total fissure area increases from 16,638 pixels to 17,707 pixels, and the total length from 2217 to 3071 pixels, the increase is more obvious. The working face continued to advance, and the fractal dimension, length, and area of the fractures also increased steadily. Until the working face advanced to 212 cm, the key layer was broken and the underlying rock layer was further compacted. The fractal dimension of the fracture was reduced from 1.56 to 1.5, the total area of the fracture was reduced from 31,451 pixels to 29,089 pixels, and the total length was increased from 5657 to 6619 pixels. During the subsequent advancement of the working face, the fractal dimension of the fracture, the total length, and the total area of the fracture gradually increased, and the change was stable.

#### Fractal dimension, length and area of local fissures

**Figure 9:**
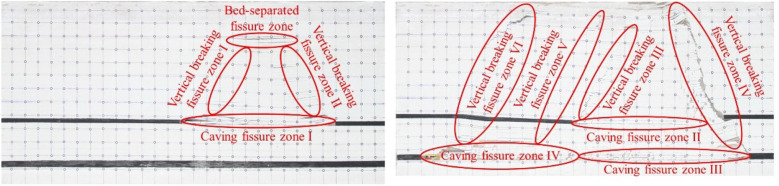
Local fissure zone division.

After the coal seam mining is completed, the fissure zone is divide according to the classification of the caving fissure zone, the bed-separated fissure zone, and the vertical breaking fissure zone, and the local fissure zone shown in Fig. 9 is obtained. Using the same processing method as above, image processing is performed independently on each fracture area, and the fractal dimension value and geometric parameters of each area are obtained. Statistics are shown in Tables 2 and 3.

**Table 2:** Classification of fissure zone after the completion of 2–2 coal seam mining.

Fissure zone division	Total fissure length/pixel	Total fissure area/pixel	Percentage of fissure (%)	Fractal dimension	Correlation coefficient
Bed-separated fissure zone	353	1103	0.12	1.2801	0.9971
Caving fissure zone I	574	3124	0.33	1.3159	0.9928
Vertical breaking fissure zone I	169	363	0.04	1.1056	0.9937
Vertical breaking fissure zone II	530	2320	0.25	1.2854	0.9943

**Table 3:** Classification of fissure zone after the completion of 3-1 coal seam mining.

Fissure zone division	Total fissure length/pixel	Total fissure area/pixel	Percentage of fissure (%)	Fractal dimension	Correlation coefficient
Caving fissure zone II	403	1683	0.18	1.2738	0.9943
Caving fissure zone III	960	4878	0.52	1.4111	0.9917
Caving fissure zone IV	1411	10,449	1.11	1.5577	0.9927
Vertical breaking fissure zone III	301	1447	0.15	1.2405	0.9938
Vertical breaking fissure zone IV	1865	14,697	1.57	1.5310	0.9951
Vertical breaking fissure zone V	185	648	0.07	1.1515	0.9954
Vertical breaking fissure zone VI	293	1662	0.18	1.2194	0.9945

The results of local fissure quantification show that the fractal dimension value, fissure length, and area of different fissure zones are quite different. Compared with the 2–2 coal seam, after the mining of the 3-1 coal seam, the enriched area of fissures increased, and the distribution of the fissure zone also changed. Since the fissure field has penetrated the surface, no longer exists in the overburden as a zone of bed-separated fissure. The caving fissure zone I transformed into the caving fissure zone II, the fractal dimension decreased from 1.3159 to 1.2738, and the length and area of the fissures also decreased, indicating that the number of fissures in the upper coal seam caving zone was reduced and the strata were further compacted. The vertical breaking fissure zones I and II are transformed into III and IV, and the fractal dimension, the length, and the area of the fractures all increase greatly. The area with the most fissures is the caving fissure zone IV, that is, the goaf behind the 3-1 coal seam stop line, and the fractal dimension is 1.5577.

### Discussion

The above studies show that the development and evolution of fractures are directly related to the change of the stress field and the structure of the overlying rock^30–32^. After the self-mining behavior occurs, along with the unloading and stress transfer of the surrounding rock mass, in the elastic stage, the rock mass deforms and produces microcracks. When the elastic limit is exceeded, the large-scale rock mass is in a plastic state or a failure state, and macroscopic cracks and fissures appear in the surrounding rock body and continue to expand. With the further increase of the mining space, the overlying rock body breaks periodically, and the mining fissures continue to develop upward in space, while in the middle of the goaf, the mining fissures are gradually compacted and closed. Throughout the mining process, the evolution of cracks follows a cyclical evolution with the periodic displacement and rupture of the overlying strata.

#### The effect of support pressure on fracture development in close coal seam mining

**Figure 10:**
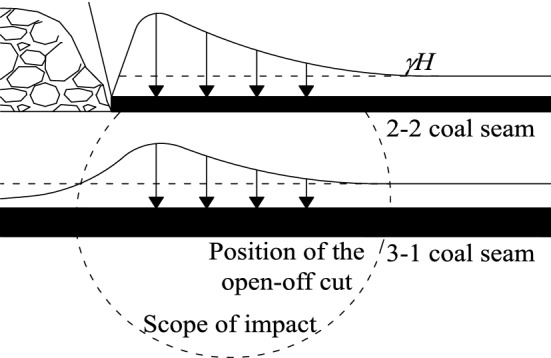
Distribution of lateral support pressure in the open-off cut.

The results of similar simulation experiments indicate that during the short-range mining process, although the gobs of the two coal seams are not connected, the overlying fissures in the gobs of the 2–2 coal seam will continue to develop upward under the influence of mining. According to the classical theory of predecessors, after the mining of the 2–2 coal seam is completed, the supporting pressure of the overlying strata will be transmitted downward through the coal wall, and the surrounding rock stress field of the 3-1 coal seam will be changed, as shown in Fig. 10. In the diagram *γ* is the bulk density of the rock and *H* is the depth of burial of the coal seam. Since the 3-1 coal seam incision is just below the stress concentration of the 2–2 coal seam incision, the mining of the 3-1 coal seam will inevitably lead to the pressure relief of the roof stratum, which will weaken the rock support capacity of the 2–2 coal seam goaf. The equilibrium state of the overlying rock is broken, and the fracture zone continues to develop upward.

#### Mining overlying rock structure under the close coal seam goaf

The previous research shows that the mining stage under the goaf of the coal seam is the most complex during the fracture evolution process^33–35^, and other mining stages are no different from the single coal seam mining. Therefore, it is the key to study the evolution of fractures to study the migration law of the overlying rock in the mining area.

**Figure 11:**
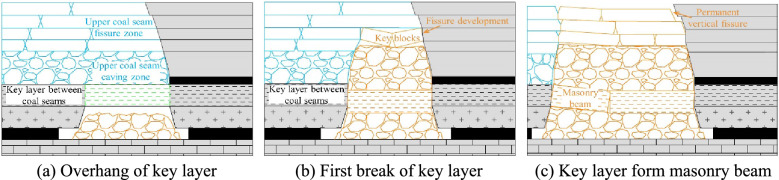
Overlying structures mined in close coal seams.

In the early stage of close coal seam mining, after the working face is fully mined, the key layer between the coal seams is in a suspended state, and it forms two supporting points of the rock beam with the coal wall. Figure 11a depicts the support beam model. As the working face advances, the key layer fails when it reaches the limit span. Since the overlying rock in the gob area of the upper coal seam is relatively broken, the broken rock layer will collapse synchronously with the key layer, and the collapse will not stop until the upper coal seam rock fissure zone. The regional distribution of the secondary caving rock formation is close to the arch, and the rock blocks at the top are in a hinged structure, as shown in Fig. 11b. After that, the working face continued to advance, and the key layer broke again after reaching the ultimate strength. The broken rock block rotated to form a "masonry beam" structure, and the overlying rock layer also collapsed synchronously again. Figure 11c shows the buckled and closed hinged rock block. It is worth noting that due to the expansion of the rock after breaking, the original fracture zone has a small displacement and a small degree of rotation during the secondary caving, The rock integrity was well preserved and few new fractures occurred. These rock formations closed again during the process, and the number of fractures did not increase. After that, the key layer enters the periodic breaking stage, and the "masonry beam" structure moves forward periodically following the advancement of the working face.

**Figure 12:**
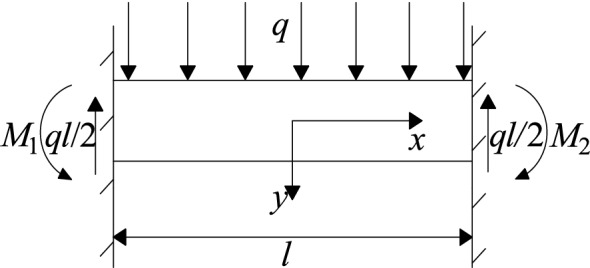
Mechanical model of solid support beam.

Figure 12 shows the simplified mechanical model of "clamped beam". The stability of the "clamped beam" is related to its maximum subsidence deformation. The tensile strength criterion is used as the failure criterion of the rock layer, and the deflection equation of the rock layer under the critical state of failure is is the formula:

2 $$L_{c} = h\sqrt {\frac{{2\sigma_{t} }}{q}}$$

3 $$I = \frac{{bh^{3} }}{12}$$

4 $$\omega (x) = \frac{q}{24EI}(x^{2} - \frac{1}{4}L_{c}^{2} )^{2}$$

where *L*_*c*_ is the length of the first broken block, m; *b* is the width of the rectangular section of the beam, m; *I* is the moment of inertia of the rectangular section, m^4^; *ω* is the deflection of the beam, m; *E* is the elastic modulus of the rock formation, MPa.

When *x* = 0, the bending subsidence deformation of the clamped beam reaches the maximum value.

**Figure 13:**
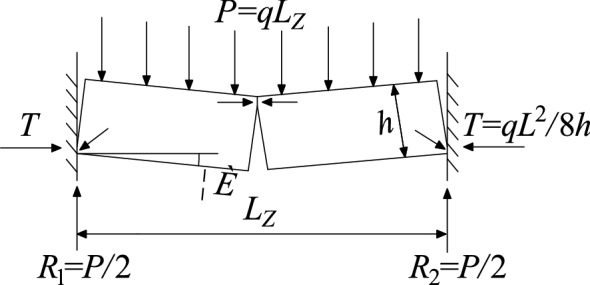
Mechanical model of articulated rock blocks.

After the key layer is broken for the first time, the upper broken rock layer collapses synchronously and will not stop until the rock layer forms a stable hinged structure. The simplified mechanical model of the hinged structure is shown in Fig. 13. The hinged structure is composed of two key rock blocks, which rotate to a certain extent during the collapse of the underlying rock formation, forming a stable mechanical structure. In the diagram *P* is the load of the rock layer above the key rock block, *T* is the force on the horizontal direction of the key rock block, *R* is the supporting force on the fulcrum at both ends of the structure, *h* is the thickness of the key rock block, *Lz* is the length of the two rock blocks before rotation, *θ* is the rotation angle of the key block. As long as the two key rock blocks do not "slip and lose stability" nor "swing instability" during the rotation process, they can form stably, preventing the rock formation from continuing to collapse until the key layer below breaks again.

**Figure 14:**
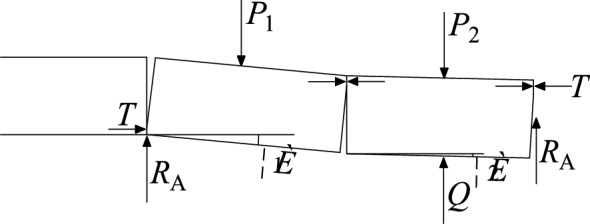
Masonry beam structural model.

After that, the key layer continued to break to form a "masonry beam" structure. The mechanical model is shown in Fig. 14. According to the "S-R" stability theory, the "masonry beam" does not experience sliding failure conditions:

5 $$i \le \tan \varphi + \frac{3}{4}\sin \theta$$

"Masonry beam" does not experience rotational instability conditions:

6 $$\frac{P}{{(i - \frac{1}{2}\sin \theta )(h - L_{Z} \sin \theta )}} \le 0.3\sigma_{c}$$

where *i* = *h/L*_*Z*_, *L*_*Z*_ is the length of the "masonry beam" rock block, m; *h* is the thickness of the rock layer, m; *φ* is the friction angle inside the broken block; *θ* is the rotation angle of the key block, °; *P* is the load of the "masonry beam", MN; *σ*_*c*_ is rock compressive strength, MPa.

#### Influence of rock left in short-distance coal seam mining hollow area on the overburden movement

In the process of short-distance coal seam mining, the direct roof rock bends and produces tensile deformation and separates from the upper part of its rock layer, which breaks into rock pieces of different sizes and fills the mining void area irregularly. After the direct roof seam collapses and fills the mining area, its volume increases due to fragmentation, resulting in the gradual weakening of the movement of the upper part of the seam. However, when the coal seam with a larger dip angle is mined downward, the downhill part of the coal seam continues to be mined to form a new mining void, and the collapsed rocks in the upper part of the mining void may slide down and fill the new mining void, so that the movement of the rock seam located in the uphill part of the mining void intensifies. According to the analysis of previous research^2,36^, leaving the rock in the quarry area can reduce the surrounding rock stress, and using waste rock to fill the quarry area mining can support the overlying rock layer in the quarry area in time, stop and resist further deformation of the surrounding rock, and prevent large displacement of the overlying rock and surface subsidence.

The above research on the development and evolution law of overburden mining fissures in shallow-buried and short-distance coal seam mining is of great significance to grasp the law of wind leakage from fissures in mining areas during mining of shallow-buried and short-distance coal seam groups, accurately determine the change in the range of coal spontaneous combustion hazard areas in mining areas affected by wind leakage, and scientifically prevent and control coal spontaneous combustion in composite mining areas of shallow-buried and short-distance coal seam groups. It is also of great significance in engineering practice applications such as coal mining control, pressure relief gas extraction, coal seam floor burst water prevention and control.

## Conclusions

In this study, the similarity simulation experiment was used to simulate the migration characteristics of overlying strata in shallow-buried and short-distance seam mining, and the development degree of mining-induced fractures in overlying strata in shallow-buried and short-distance seam mining was quantitatively described by using fractal dimension through image processing technology, which made an original contribution to the study on the dynamic evolution law of mining-induced fractures in shallow-buried and short-distance mining. The conclusions are as follows:

1. Similar simulation results show that the overlying fracture field, displacement field and stress field will evolve synchronously with the advancement of the working face, and the evolution process is most complicated in the mining stage under the goaf; the different displacements of the adjacent rock formations above and below will produce bed separation cracks, and the same rock formation will produce vertical fracture fractures with different displacements. In the mining stage of the gob, only after the key stratum between the coal seams is broken, the stress value of the overlying strata of the upper coal seam will change significantly, and the overburden stress of lower coal seam will also have a leaping change due to its breaking.
2. The results of fissure quantification show that in the mining stage under the goaf, the fractal dimension of the fissures increases from 1.4 to 1.5 after the key layers between the coal seams are broken, the total area of fissures increases from 16,638 pixels to 17,707 pixels, and the total length increases from 2217 to 3071 pixels; after the main key layer of the overlying rock is broken, the fractal dimension of the fractures is reduced from 1.56 to 1.5, the total area of the fractures is reduced from 31,451 pixels to 29,089 pixels, and the total length is increased from 5657 to 6619 pixels. In other mining processes, the fractal dimension of the fracture basically increases stably. The fractal dimension value of each local fracture area is also quite different after the mining is completed.
3. In the process of mining under the goaf, the overlying rock structure mainly undergoes three changes, namely, the key layers between the coal seams are suspended and leaked to form "fixed beams," which isolate the fissure field of the upper and lower coal seams; during the first caving stage of the key layer, a large number of fissures were formed to penetrate the goaf of the upper and lower coal seams, and the rock blocks in the original fissure zone formed a hinged structure to prevent the strata from continuing to collapse; after that, the key layer periodically collapsed to form a "masonry beam" structure; and the overlying rock synchronously subsided to form a "masonry" structure; fissures are not produced in large numbers.

## Data Availability

All data generated or analyzed during this study are included in this published article.
